# The need for, feasibility of, and barriers to implementation of a trauma traveling fellowship: a survey of ESTES members

**DOI:** 10.1007/s00068-026-03321-2

**Published:** 2026-09-01

**Authors:** Jared M. Wohlgemut, Shailvi Gupta, Stefano P. B. Cioffi, Heura Llaquet-Bayo, Helen S. Wohlgemut, Jonathan Hobby, Shahin Mohseni, Diego Mariani, Christine Gaarder, Gary A. Bass, Susan Brundage

**Affiliations:** 1 Centre for Trauma Sciences, Blizard Institute, Queen Mary University of London, London, UK; 2 R Adams Cowley Shock Trauma Center, University of Maryland Medical Center, Baltimore, USA; 3 ASST Grande Ospedale Metropolitano Niguarda, Milan, Italy; 4 Hospital Universitari Parc Taulí, Corporació Sanitària i Universitària Parc Taulí, Sabadell, Barcelona, Spain; 5 School of Health and Wellbeing, University of Glasgow, Glasgow, UK; 6 Department of Orthopaedic Surgery, Hampshire Hospitals NHS Foundation Trust, Basingstoke, UK; 7 Trauma & Acute Care Surgery, Department of Surgery, New Jahra Hospital, Al Jahra, Kuwait; 8 School of Medical Sciences, Orebro University, Orebro, Sweden; 9 ASD Urgenza e Trauma ASST OVEST MILANESE, Legnano, Milan, Italy; 10 Department of Traumatology, Oslo University Hospital, University of Oslo, Oslo, Norway; 11 Division of Traumatology, Surgical Critical Care & Emergency Surgery, Department of Surgery Perelman School of Medicine, University of Pennsylvania, Philadelphia, USA; 12 Center for Clinical Epidemiology and Biostatistics, Perelman School of Medicine, University of Pennsylvania, Philadelphia, USA

**Keywords:** Trauma surgery, Fellowship, Survey, European, Implementation

## Abstract

**Purpose:**

Many European surgeons lack access to high-volume trauma training. A Trauma Traveling Fellowship, supported by the European Society of Trauma and Emergency Surgery (ESTES), could broaden educational and clinical exposure to trauma care, but feasibility and implementation factors are unclear. The aims of this survey are to assess the aspirations, expectations, and perceived challenges of prospective fellows and host centres for an ESTES Trauma Traveling Fellowship.

**Methods:**

A cross-sectional, web-based survey was distributed to all ESTES members, capturing demographics, fellowship aspirations, perceived barriers, and host centre capacities. The survey incorporated Likert scales, free-text responses, and was guided by the Consolidated Framework for Implementation Research (CFIR) and the Reach, Effectiveness, Adoption, Implementation, and Maintenance / Practical Robust Implementation and Sustainability Model (RE-AIM/PRISM).

**Results:**

Of 903 invited, 210 responses (23.3%) were analysed. The median age was 38 years; 71% were consultants, and 29% were residents. Most (71%) would consider applying for a Fellowship, and 47% stated their centre could host Fellows. Only 23% currently access funded fellowships. Key aspirations included hands-on participation in trauma care, although hosts could more often only guarantee observational roles. Major barriers included funding and institutional permissions. Most respondents supported implementing the fellowship as it was perceived to align with ESTES priorities.

**Conclusions:**

There is strong support among European trauma surgeons for an ESTES Trauma Traveling Fellowship, recognising both its potential value and practical impediments. Addressing funding and regulatory barriers will be crucial. A structured and adaptable implementation approach, combined with ongoing evaluation, may help ensure long-term success.

**Supplementary Information:**

The online version contains supplementary material available at https://doi.org/10.1007/s00068-026-03321-2.

## Introduction

Trauma surgery has been called a “discipline in crisis” due to logistical, political, and workforce challenges [1]. A key logistical and workforce challenge has been an increasing shift to nonoperative management, resulting in less operative exposure for residents and surgeons [1]. There is significant international variation in mechanisms of injury and operative volume, as well as in trauma training and workforce structures, all of which affect surgeons’ ability to obtain adequate training to manage complex injuries [2–4]. Trauma fellowship programs have become an important way to improve trauma operative and non-operative skills [5–7].

Exposure to centres of excellence in trauma care can enhance surgeons’ capacity to manage complex injuries and may encourage early-career surgeons to pursue trauma surgery as a formal speciality [7]. Each nation has a unique trauma system, as well as nuance in designating trauma centres, such as the American College of Surgeons trauma center verification programme (https://www.facs.org/quality-programs/trauma/quality/verification-review-and-consultation-program/about-vrc/), and the United Kingdom network of regional major trauma centres supported by trauma units (https://digital.nhs.uk/about-nhs-digital/corporate-information-and-documents/directions-and-data-provision-notices/data-provision-notices-dpns/national-major-trauma-registry-nmtr). There is no international mechanism to designate institutions as centres of excellence in trauma care, so no exhaustive list of centres of excellence in trauma care exists. However, many trauma centres have an international reputation, either because of high-volume caseload, high quality care, or academic/educational excellence, which prospective fellows may wish to experience. Despite the fact that surgeons of various subspecialties frequently encounter trauma patients, many receive limited experience with high-volume trauma centres during their training or early employment [3]. Financial and logistical barriers may prevent them from accessing such immersive experiences. A dedicated Trauma Traveling Fellowship could address these challenges by facilitating structured placements and funding opportunities, thereby enabling surgeons to improve their trauma decision-making, contribute to educational and/or research activities, and potentially learn specialised skills in trauma care. International fellowship programs vary by surgical specialty, duration and funding structure, and can facilitate exposure to diverse healthcare systems, knowledge exchange, and innovative clinical practices [8–10]. Programs have been designed to focus on academic partnership, education, clinical skills, and military-civilian exchange [11–13].

The European Society of Trauma and Emergency Surgery (ESTES) is committed to promoting interest, knowledge, and quality in emergency and trauma surgery (https://www.estesonline.org). While its annual congress, training courses, and other sponsored events fulfil much of this mandate, a Trauma Traveling Fellowship may extend ESTES’ educational impact by offering personalised, experiential learning in advanced trauma settings, defined by either accreditation, volume and/or clinical excellence. Other societies, such as the Federation of European Societies for the Surgery of the Hand (FESSH) (https://fessh.com/travel-award/) [14], the World Society of Emergency Surgery (WSES) (https://www.wses.org.uk/news/wses-fellowship-in-emergency-surgery-and-trauma-2025), and the European Society of Shoulder and Elbow Surgery (SECEC-ESSSE) (https://www.secec.eu/education/fellowship/intraeuropean-travelling-fellowship) have demonstrated the feasibility of traveling fellowships in related surgical fields, but it remains unclear whether a similar model would effectively meet the specific needs of trauma surgeons across Europe. In addition, many surgical subspecialties may be involved in trauma care, thus any fellowship should accommodate this. As ESTES is a society that was a historic amalgamation between two societies focusing on orthopaedic trauma, and visceral/general trauma, an ESTES trauma fellowship would likely focus on either orthopaedic trauma, visceral trauma, or both, depending on the context of the host institution’s capabilities and the prospective fellow’s capabilities and interests.

Formal fellowships in trauma or acute care surgery do not exist in many European countries, even though some residency programs allow short-term external rotations. A traveling fellowship cannot fully replicate a structured postgraduate program, but it may meaningfully expand the training landscape for individuals considering a long-term focus on trauma surgery. There is also a need to broaden trauma surgical competence in view of the increased preparedness situation, and trauma fellowships are one way to address this gap. In a recently published survey of 123 ESTES members across 20 European countries, the initiative identified traveling clinical fellowships as the highest priority for fostering mentorship in trauma [15]. Two implementation frameworks- the Consolidated Framework for Implementation Research (CFIR; https://cfirguide.org) and the Reach, Effectiveness, Adoption, Implementation, and Maintenance / Practical Robust Implementation and Sustainability Model (RE-AIM/PRISM; https://re-aim.org) - offer valuable insights for designing, delivering, and sustaining this proposed initiative [16]. CFIR identifies context-specific facilitators and barriers during the implementation phase, while RE-AIM evaluates the broader reach, impact, and long-term viability of an intervention. By applying both frameworks, it becomes possible to examine how a Trauma Traveling Fellowship can be adopted and integrated across diverse settings, and whether it can be maintained as a core educational tool within ESTES.

The present survey aims to assess the aspirations, expectations, and perceived challenges of prospective fellows and host centres for an ESTES Trauma Traveling Fellowship, guided by CFIR and RE-AIM/PRISM constructs.

## Methods

### Design

A cross-sectional survey was developed by the Visceral Trauma Section (VTS) of ESTES using the Survey Monkey platform (https://www.surveymonkey.com). The instrument underwent pilot testing by ESTES executive leadership (S.G., S.P.B.C., H.L.B., S.M., G.A.B, and S.B.), who offered feedback on the phrasing of questions, the overall structure, and the time required for completion. Revisions were made accordingly, and the final survey required an average of five minutes to complete. The questionnaire included demographic items, Likert-type scales, and free-text responses, each pertaining to the anticipated benefits and challenges of a proposed Trauma Traveling Fellowship. In the survey, participants were asked two questions to ensure we obtained answers from prospective fellows, and prospective host institutions. If the answer to the first question (“Would you be interested in applying for an ESTES Trauma Traveling Fellowship?”) was yes, then the questions pertaining to prospective fellows appeared in the survey. If the answer to the second question (“Would your centre/hospital be willing to host a Trauma Traveling Fellow?”) was yes, then the questions pertaining to prospective hosts appeared in the survey.

### Approvals and consent

This survey did not involve patient data and therefore qualified for exemption from full ethical or Institutional Review Board review. Data collection adhered to the General Data Protection Regulation (GDPR). The survey design, distribution, and reporting followed the Checklist for Reporting Results of Internet E-Surveys (CHERRIES) guidelines [17].

A dedicated introductory statement informed participants of the survey’s aims, purpose, approximate duration, and data management protocols. Responses were anonymised and stored on a secure server. The statement further identified the VTS of ESTES as the overseeing entity and disclosed that aggregate findings would be submitted for peer-reviewed publication. Participants were required to indicate consent to proceed, though no incentives were offered.

### The distribution

The survey link was disseminated via email to all registered ESTES members via the ESTES administrative management company (Conventus) on 13th April 2025, thus constituting a closed survey. A poster containing a QR code was displayed at the VTS meeting in 13–15 April 2025 to encourage further participation. A reminder email was sent two weeks later (26th April 2025), as part of an ESTES newsletter email on 10th June, and a final dedicated email on 16th June 2025. The survey was closed on 18th July 2025, approximately 3 months after it was opened.

### Statistical methods

Response rates were computed by comparing the number of completed submissions to the total invitations issued. Descriptive statistics were employed, with continuous variables reported as means and standard deviations or medians and interquartile ranges, depending on distribution (using the D’agostino -Pearson test), and categorical variables expressed as frequencies and percentages. CFIR-related questions were coded as potential barriers or facilitators, with an assessment of their impact on fellowship implementation. RE-AIM items relied on a six-point Likert scale, and free-text entries were obtained, with a plan to evaluate them using inductive qualitative analysis to elucidate additional nuances and contextual factors. Maps were produced on mapchart.net, and statistical analyses and graphs were produced on Graphpad Prism 10 for macOS v10.4.0 (Graphpad Software LLC, Boston, MA, USA). To ensure there were no duplicate responses, conditional formatting on Microsoft Excel v16.92 (Microsoft Corp, Redmond, WA, USA) was used to identify any duplicate IT addresses submitted. Of these, there were 19 IP addresses identical to each other, and five pairs of two. The demographics were evaluated and were sufficiently dissimilar, such that each response was considered a unique participant response.

## Results

**Fig. 1:**
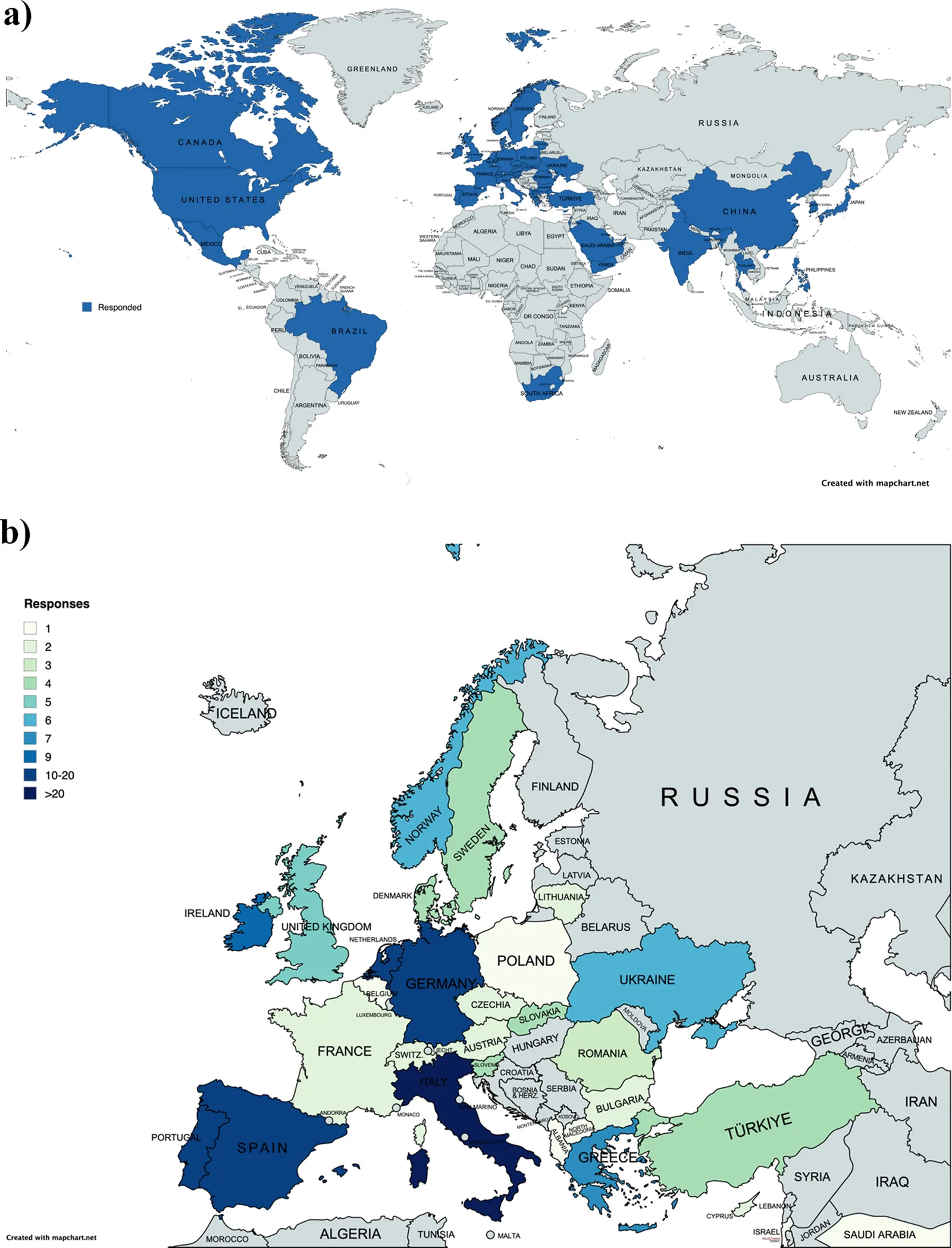
Maps of Contributing Countries, (**a**) World map and (**b**) European map

A total of 903 participants were invited to take part in the survey, of which 244 participants started the survey. From those, 210 participants completed the survey and were included in the final analyses (response rate 23.3%). Demographics are presented in Table 1, and Fig. 1. The cohort comprised 39% females with an overall median age of 38 years (IQR 32–47). The majority were consultant trauma surgeons (71%), while over one quarter were surgical residents (28%). Career stage was distributed relatively evenly among the respondents, 28% being in residency at the moment of the survey, 21% having 1–5 years of post-training experience, 25% having 6–15 years of post-training experience, and the remaining 26% having more than 15 years of post-training experience. Respondents stated that their surgical specialties (respondents could choose more than one) included: 64% trauma surgeons, 78% emergency general surgeons, 13% orthopaedic surgeons, and 26% were from other surgical specialties. Baseline questions are presented in Table 2.

**Table 1:** Demographics (*n* = 210)

Demographic	Variable	Missing	Number	%
Age (Median, IQR)		0	38 years	(32–47 years)
Sex	Female		82	39.0%
Role in Hospital	Resident in Training	0	58	27.6%
	Attending/Consultant		149	71.0%
	Other*		3	1.4%
Career Stage	Surgical resident	3	58	27.6%
	Early career (1–5 years post-training)		45	21.4%
	Mid-career (6–15 years post-training)		52	24.8%
	Late career (> 15 years post-training)		55	26.2%
Surgical Specialty	Trauma Surgery	0	134	63.8%
	Emergency General Surgery		164	78.1%
	Orthopaedic Surgery		27	12.9%
	Other**		54	25.7%

*Including two medical students and one physician assistant

**Including general surgery (15), abdominal wall surgery (5), colorectal surgery (5), upper gastrointestinal surgery (5), oncological surgery (4), surgical critical care (3), bariatric surgery (3), thyroid/endocrine surgery (2), gastrointestinal surgery (2), vascular surgery (2),hepato-pancreatico-biliary surgery (2), plastic surgery (1), laparoscopic surgery (1), burn surgery (1), paediatric surgery (1), emergency medicine (1), thoracic surgery (1)

**Table 2:** Baseline questions

Question	Response	Missing	Number	%
Do you currently have access to externally funded visiting rotations/fellowships?	Yes	9	48	22.9%
Have you previously undertaken an externally funded rotation/fellowship?	Yes	9	62	29.5%
What would be the ideal duration of a Trauma Traveling Fellowship?	1–2 weeks	9	9	4.3%
	3–4 weeks		35	16.7%
	1–2 months		47	22.4%
	3–6 months		78	37.1%
	6–12 months		32	15.2%
Would you be interested in applying for an ESTES Trauma Traveling Fellowship?	Yes	9	149	71.0%
	No but I think it is an interesting idea		49	23.3%
	No and I do not think it is necessary		3	1.4%
Could you personally (or could your institution) contribute funds to support such a Fellowship?	Yes	24	92	43.8%
	No		94	44.8%
Would your centre/hospital be willing to host a Trauma Traveling Fellow?	Yes	28	98	46.7%
	No but I think it would be interesting in other centres		64	30.5%
	No and I do not think it is necessary		20	9.5%
Does your hospital or department have any capacity to sponsor or partially fund a Traveling Fellow?	Yes	6	24	11.4%
	No		180	85.7%

**Fig. 2:**
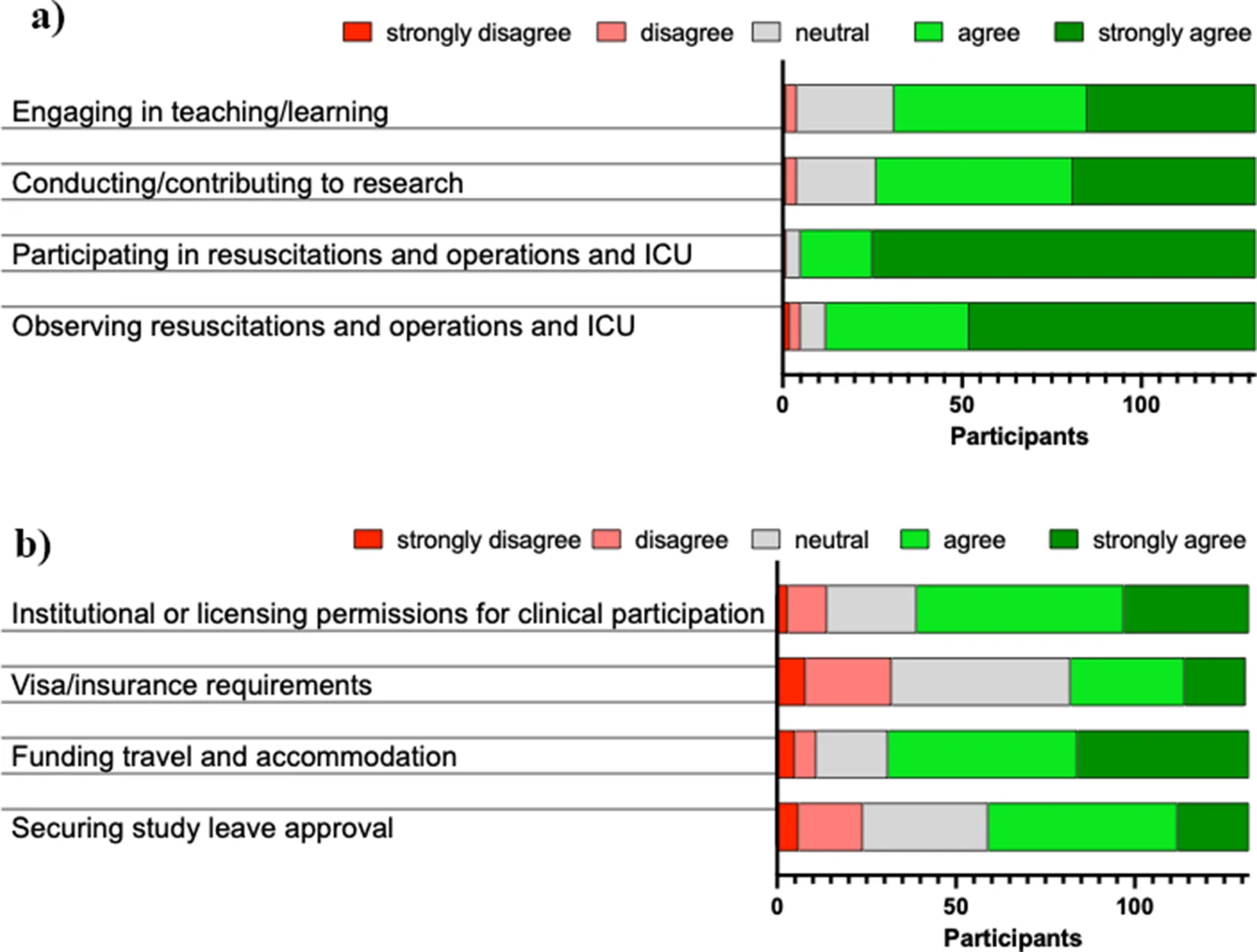
Responses from prospective fellows, including: (**a**) fellowship aspirations (“what experiences would you hope to gain as a traveling fellow?”), and (**b**) fellow barriers (“what do you foresee as the greatest barriers to attending a Traveling Fellowship?”) (*n* = 132; 17 missing)

Most participants agreed that they would like to participate in resuscitations and operations and ICU, rather than simply observe these activities (Fig. 2a). Still a majority were interested in conducting or contributing to research and engaging in teaching and learning. The greatest barriers to attending a traveling fellowship were funding travel and accommodation, followed by institutional or licensing permissions for clinical participation, securing study leave approval and lastly, visa/insurance requirements (Fig. 2b). The three most commonly mentioned preferred fellowship sites in Europe are within the UK, Norway, and Germany (Table S1). The three most-preferred fellowship sites in countries other than Europe include the USA, South Africa, and Colombia (Table S2).

**Fig. 3:**
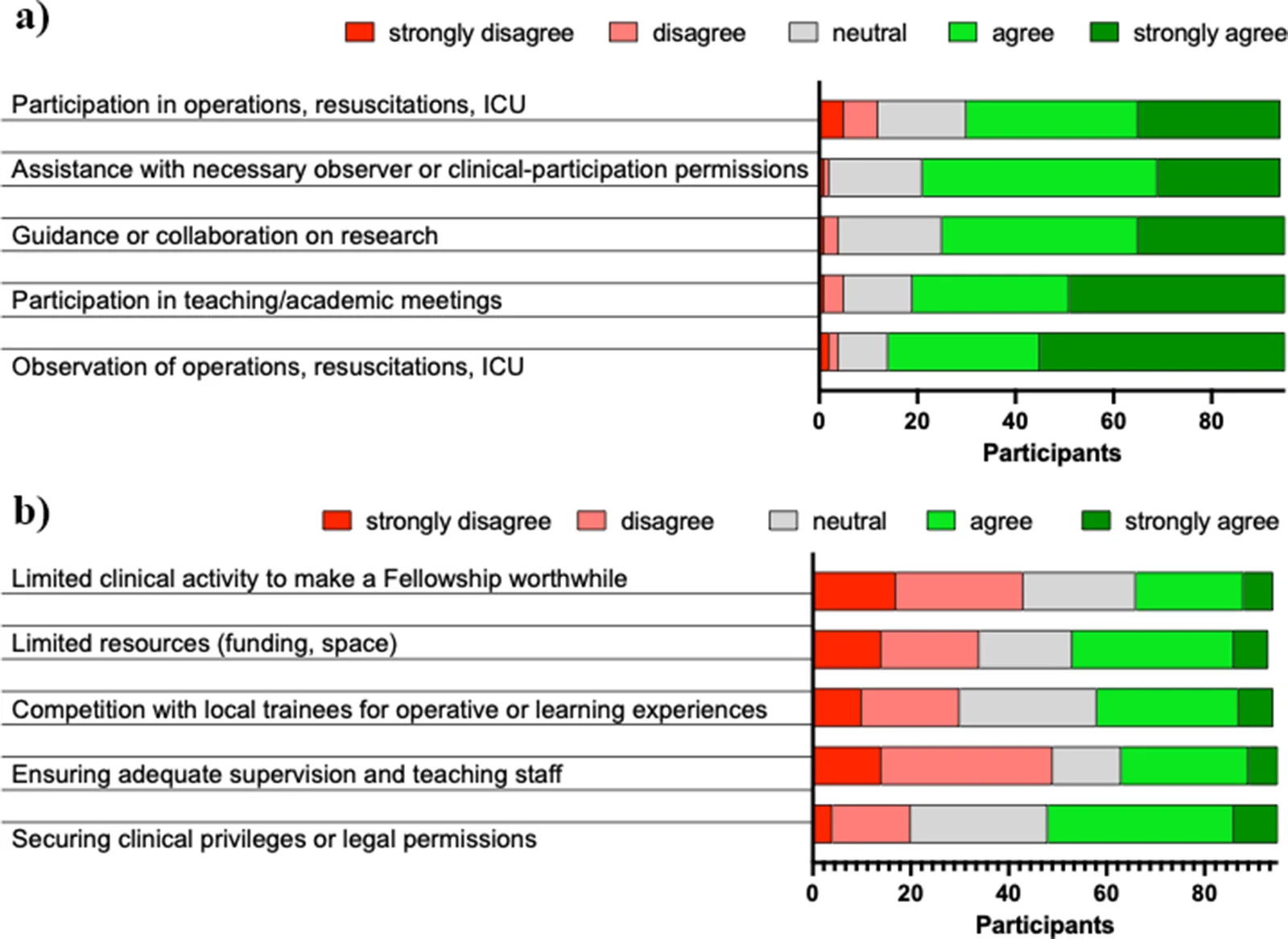
Responses from prospective hosts, including: (**a**) host expectations (“what opportunities could your centre realistically offer to a Traveling Fellow?”), and (**b**) host barriers (“what potential barriers might prevent your centre from hosting a Traveling Fellow?”) (*n* = 95; 3 missing)

A majority of hosts expected that they would be able to provide potential traveling fellows with observational experience (operations, resuscitation, and ICU), participation in teaching/academic meetings, guidance on collaboration or research, assistance with necessary observer or clinical-participation permissions, and participation in operations, resuscitation, and ICU (Fig. 3a). The barriers that hosts might face were securing clinical privileges or legal permissions for fellows, followed by limited resources (funding, space), competition with local trainees for operative/learning experiences, ensuring adequate supervision and teaching staff, and limited clinical activity (Fig. 3b).

Following the CFIR methodology, the vast majority of respondents agreed that the Traveling Fellowship is a needed initiative which aligns with ESTES leadership goals and priorities (Figure S1). Over half agreed that we regularly use performance or feedback data, and that ESTES has sufficient resources to implement a fellowship. Less than half agreed that they have good communication with key ESTES stakeholders.

Following the PRISM methodology, the majority of respondents agreed (either “largely” or “completely”) that an ESTES Trauma Traveling Fellowship would align with potential fellow’s expectations, that it would align with European trauma practice, that it would align with the expectations of the Host Centres, and that it would align with available Host Centre resources (Figure S2).

Following the RE-AIM methodology, well over half agreed (either “largely” or “completely”) that this Fellowship would measurably improve trauma training for Fellows, that a wide variety of fellows will apply for this fellowship, that a high percentage of eligible fellows will apply and complete a fellowship, and that participating sites will consistently offer core experiences (Figure S3). Approximately half agreed (either “largely” or “completely”) that an ESTES fellowship would be sustained over 1–2 years, that a wide variety of centres would become Host Centres, and that a high percentage of invited host centres will host a fellow.

There were very limited free text responses, including three from CFIR questions, six for PRISM questions, and nine for RE-AIM questions, precluding meaningful inductive qualitative analysis.

## Discussion

The main finding of this study is the widespread perceived need for an ESTES Trauma Traveling Fellowship, along with a broad interest in either applying as a fellow, or offering to be a host centre. Specific aspirations and anticipated challenges were identified by prospective fellows, as well as expectations and anticipated difficulties for prospective host centres.

This study has several implications related to its more detailed findings. First, there is a discrepancy between what fellows find most valuable – participating in resuscitations, operations, and ICU – and what host centres can realistically provide due to national or local regulations, with approximately 9 in 10 host centres stating they are unable to provide coverage for hands-on/patient-contact experience. Institutional or licensing permissions for clinical participation were anticipated to be a significant barrier to attending a trauma fellowship, acknowledged by prospective fellows and host centres alike. Fellows and host centres may be able to address this by understanding how individual fellows are granted permissions in the host country, and supporting prospective fellows with their applications. The European Board of Surgery Qualification (EBSQ; https://uemssurg.org/whatwedo/ebsq-examinations/) - a Europe-wide qualification which ensures a high surgical examination standard – may help the licencing process for fellows. For instance, a fellow with the EBSQ may be permitted to treat patients and perform operations, but this would have to be agreed by the host institution and host nation.

Second, the largest barrier acknowledged by prospective fellows is funding for travel and accommodation, and the second largest barrier acknowledged by host centres is also limited financial resources. Clearly, money is required for this initiative- and will have to be specifically sourced and budgeted prior to launch of this fellowship, as only just over half of respondents perceived that ESTES has sufficient resources to implement the fellowship. One potential solution to this funding problem is for national institutions to support this initiative with grants, thereby reducing the funds required from the international organisation [14].

Third, using CFIR methodology, the vast majority of participants stated that ESTES should implement the Trauma Fellowship, that it is needed, and that it aligns with ESTES goals. The implication of these findings is that there is impetus to prioritise this initiative from within the organisation, as members clearly value it. An unexpected finding was that less than half of the respondents agreed they had good communication with key ESTES stakeholders. Though not the aim of this study, this finding can be used to highlight organisational aspects of ESTES.

Fourth, respondents stated that they expect to contribute to an academic or educational project, alongside clinical experiences, during the fellowship. Having the fellow contribute to academic activities, including peer-reviewed publication and presentation, would further increase the benefit of the fellowship to the fellow’s career, as well as enrich the scientific network who will help fund the fellow.

Fifth, though the absolute differences between responses were small, there was more agreement that a wide variety of fellows will apply for the fellowship, than a wide variety of host centres will host them. This is not surprising, as not all hospitals or institutions will have enough trauma volume to support visiting fellows, yet trainees from a wide variety of settings may be interested in applying to improve their training.

Sixth, anecdotally, the authors acknowledge that, generally, fellowships in some high-volume but low-resource countries may permit more hands-on clinical exposure than in other countries with lower volumes and higher resources, because regulatory permission processes may differ. However, there may be differences and nuances in trauma management between settings, which could lead to discrepancies between the experience fellows have during their fellowship and the expectations of patient management when they return to their home countries afterwards. The authors feel it is important to learn local medico-legal processes and respect clinical and cultural practices while on fellowship, but also be cognizant upon return to their home countries that not all practices are identical or acceptable globally, and to also learn local medical-legal processes and respect clinical and cultural practices at home. This could be addressed, for example, by an on-boarding / induction session on the first day of the fellowship by the host institution, introducing the fellow to specific medico-legal, clinical, and organisation aspects of the institution.

The proposed ESTES Trauma Traveling Fellowship will be designed to enhance both the educational and networking opportunities available to surgeons with an interest in trauma care. By creating structured rotations at selected trauma centers, the fellowship will aspire to broaden professional development and promote collaborations across institutional and national boundaries [8–11]. In contexts where formalised trauma fellowship pathways remain underrepresented, such a structured program can help bridge existing gaps in training by immersing participants in high-volume, specialised environments [1, 6–7, 12]. To guide the design and evaluation of this fellowship, we deliberately applied key implementation science frameworks. The Consolidated Framework for Implementation Research (CFIR) offers a systematic process for identifying local factors that may influence the fellowship’s feasibility and uptake, including leadership support, organisational readiness, and resource availability [16]. This approach enables targeted strategies to address recognised barriers, such as difficulties securing funding or establishing the requisite institutional agreements for hosting visiting surgeons. In parallel, RE-AIM provides a structured mechanism for assessing the fellowship’s reach, effectiveness, adoption, implementation fidelity, and maintenance. Its emphasis on iterative refinement helps ensure that both host centres and prospective fellows benefit from ongoing feedback loops. Through regular monitoring of reach and adoption, for instance, leaders can detect whether the fellowship is attracting an inclusive cross-section of learners and refine selection criteria or application processes as needed. Likewise, attention to maintenance prompts early planning for long-term sustainability, reinforcing the fellowship’s position as a durable resource within ESTES. By utilising CFIR’s context-specific questions, alongside RE-AIM’s broader approach, ESTES will be able to ensure the program is implemented in a thoughtful way, anticipating and addressing problems before they arise. As a result, the fellowship can be continually refined to address emerging needs, align with the priorities of host institutions, and sustain momentum over time. A key early issue will be how to match fellows to host institutions: either informally based on the fellow’s specialty interests, operative volume and learning goals, or through a formal match algorithm, similar to the National Resident Match Program (https://www.nrmp.org) used to match residents to surgical training programs in the USA.

The limitations of this survey are related to its cross-sectional design, which may have introduced selection bias, recall bias, or response bias. Future work will include a further evaluation during the implementation process, and a final survey after implementation and use, to determine whether the fellowship will meet the fellows’ and the host centres’ goals.

## Conclusion

This survey demonstrates widespread support for an ESTES Trauma Travelling Fellowship among European trauma surgeons and identifies the principal facilitators and barriers to its successful implementation. By addressing funding, regulatory, and organisational challenges, ESTES has an opportunity to develop a structured fellowship that complements existing educational initiatives and expands access to high-volume trauma training.

## Supplementary Information

Below is the link to the electronic supplementary material.


Supplementary Material 1


## Data Availability

This survey did not involve patient data and therefore qualified for exemption from full ethical or Institutional Review Board review. Data collection adhered to the General Data Protection Regulation (GDPR). The survey design, distribution, and reporting followed the Checklist for Reporting Results of Internet E-Surveys (CHERRIES) guidelines.
