# Supplementary material for: The need for, feasibility of, and barriers to implementation of a trauma traveling fellowship: a survey of ESTES members
Source: Eur J Trauma Emerg Surg. 2026 Sep 1;52(1):278. doi: 10.1007/s00068-026-03321-2 (PMC13534211; doi:10.1007/s00068-026-03321-2)
Supplement: Supplementary file 1 [file 68_2026_3321_MOESM1_ESM.docx]

## Supplementary Material

**Table S1.** Preferred European Fellowship sites

| Preferred European Sites | | | |
| --- | --- | --- | --- |
| Country | Mentioned | Specific Hospital | Mentioned |
| UK | 79 | Royal London Hospital  London  Kings College Hospital  St Georges Hospital  St Mary’s Hospital  Leeds  John Radcliffe Hospital  Sheffield Northern General Hospital | 31  19  4  4  3  3  2  1 |
| Norway | 39 | Oslo University Hospital Ulleval  Rural hospital | 30  1 |
| Germany | 36 | Charité Universitätsmedizin, Berlin  RWTH Aachen University Hospital  Berlin  Helios Hospital Berlin  University Hospital Munich  Hamburg  Unfallkrankenhaus Berlin (UKB)  UPMC Hannover  Frankfurt  UTZ Murnau  Klinikum rechts der Isar, Technical University of Munich  AUVA Trauma Hospital (Lorenz Böhler UKH, Вена)  Landstuhl Regional Medical Center | 5  3  3  3  2  2  2  2  2  1  1  1  1 |
| Spain | 27 | Hospital del Mar, Barcelona  Barcelona  Hospital Universitari parc Tauli, Barcelona  Hospital Universitario Vall d’Hebron, Barcelona  Universitario Gregorio Marañón, Madrid, Spain  Seville  Madrid | 11  6  1  1  1  1  1 |
| Italy | 25 | Milan  Unit at Ospedale Maggiore Policlinico, Milan  Niguarda, Milan  Rural (University Hospital Sassari)  Rizzoli Orthopaedic Institute, Bologna  Pisa  Kronplatz Itália  Cesena | 8  2  2  1  1  1  1  1 |
| Sweden | 20 | Karolinska Trauma Centre, Stockholm  Örebro University Hospital  Malmö | 14  4  1 |
| Switzerland | 17 | University Hospital Zurich  Inselspital (Bern University Hospital)  CHU de Grenoble Alpes | 13  3  1 |
| Netherlands | 15 | University Medical Center Utrecht (UMCU)  Academic Medical Center, Amsterdam  Leiden University Medical Centre  Rotterdam  Erasmus MC Medical | 4  4  3  1  1 |
| France | 11 | Grenoble Alpes University Hospital  Hospital de la Timone  Pitié-Salpêtrière  Paris  Lyon  Saint Antoine Hospital Paris | 1  1  1  1  1  1 |
| Finland | 8 | HUS Helsinki University Hospital | 5 |
| Denmark | 6 | Rigshospitalet Copenhagen | 5 |
| Austria | 4 | Vienna  Innsbruck | 2  1 |
| Portugal | 3 | Hospital de São José, Lisbon  Santa Maria Hospital, Lisbon | 1  1 |
| Poland | 3 | [no specific hospital or city mentioned] |  |
| Belgium | 2 | University Hospitals Leuven  Antwerp University Hospital | 1  1 |
| Other* | 1 |  |  |
| *Other countries mentioned once include: Portugal, Slovakia (University Hospital Bratislava), Czech Republic (St. Zdislava Hospital), Hungary, Greece (Athens), Turkey, Serbia (Belgrade), Croatia (Zagreb), Ireland, Estonia, Albania. Don’t know included 5. Other included 3, with no further comment. | | | |

**Table S2.** Preferred Non-European Fellowship sites

| Preferred European Sites | | | |
| --- | --- | --- | --- |
| Country | Mentioned | Specific Hospital | Mentioned |
| USA | 111 | R Adams Cowley Shock Trauma Center, Baltimore, MD  Massachusetts General Hospital, Boston, MA  Los Angeles County, CA  University of Chicago, IL  USC Keck Medical Center, CA  Mayo Clinic, Rochester, MN  Seattle, WA  Presbyterian Weill Cornell Medical Center, New York, NY  Johns Hopkins Hospital, Baltimore, MD  Pennsylvania Trauma Center, Philadelphia, PA  Stanford Healthcare, CA  Red Duke Trauma Institute at Memorial Hermann-Texas Medical Center, TX  Miami, FL  Maryland  New York  University of Alabama at Birmingham, AL  Cleveland Clinic, Cleveland, OH  Cincinnati, OH  Inova Fairfax Medical Campus, VA  University of Mississippi Medical Center, MS  Hospital for Special Surgery (HSS), New York, NY  Ryder Trauma Centre, Miami, FL  Jackson Memorial, Miami, FL  California  Memphis, TN  Trauma Center at NYU Langone Hospital—Long Island, NY  University hospital, Columbia, NY  Cedars-Sinai Medical Center, Los Angeles, CA  Denver Health, CO  Arizona  Yale New Haven Hospital, CT | 26  8  5  4  4  3  3  3  3  2  2  2  2  2  2  1  1  1  1  1  1  1  1  1  1  1  1  1  1  1  1 |
| South Africa | 54 | Chris Hani Baragwanath Academic Hospital, Johannesburg  Capetown  Johannesburg  Tygerberg hospital, Cape Town  Stellenbosch  Durban  Milpark Hospital, Johannesburg  Groote Schuur Hospital | 10  9  8  2  2  1  1  1 |
| Colombia | 11 | Hospital Universitario del Valle, Cali  Fundacion Hospital  Medellin Hospital, Antioquia | 4  2  1 |
| Brazil | 9 | Campinas | 3 |
| Israel | 8 | Sheba medical center, Tel Aviv  Tel Aviv Sourasky Medical Center (Ichilov)  Israel Hospital General | 3  1  1 |
| Canada | 8 | Sunnybrook Health Sciences Centre, Toronto, ON  St Michael’s Hospital, Toronto, ON  McMaster, Hamilton, ON  Montreal, QC  Vancouver, BC | 3  1  1  1  1 |
| Australia | 7 | John Hunter Hospital, Newcastle, NSW  Royal Darwin Hospital, NT | 3  1 |
| Japan | 5 | Tokyo | 1 |
| China | 3 | Beijing | 1 |
| UAE | 3 | Mediclinic Parkview hospital, Dubai  Rashid Hospital, Dubai | 1  1 |
| Korea | 3 | Asan Hospital, Seoul  Seoul National University Bundang Hospital – Seongnam, South Korea | 1  1 |
| Other* | 1 |  |  |
| *Other countries mentioned once include India, Mexico, Ethiopia. Don’t know included 6. Other included 3, with no further comment. | | | |

**Fig. S1:** CFIR questions (unabridged questions in Supplementary material). (n=174; 36 missing)

**Fig. S2:** PRISM questions (unabridged questions in Supplementary material). (n=165; 45 missing)

**Fig. S3:** RE-AIM questions (unabridged questions in Supplementary material). (n=165; 45 missing)

## Survey Questions

## Survey Initial Statement

Invitation

You have been invited to participate in this survey as a valued member of ESTES. Survey completion is voluntary, and no incentives are offered to participants.

Rationale

Visiting a centre of excellence in trauma care may improve a surgeon’s care of injured patients and motivate them to pursue a career as a trauma surgeon. However, funding and opportunities are limited for surgeons to visit centres of excellence in trauma care. A Trauma Traveling Fellowship would increase exposure to world-class trauma care and provide the opportunity and funding to allow surgeons to benefit. The mission of the European Society of Trauma and Emergency Surgery (ESTES) is to “promote interest, knowledge and quality of care in emergency and trauma surgery” (<https://www.estesonline.org/about-us/mission-statement/>). A Trauma Traveling Fellowship would help ESTES meet their stated mission statement.

Aims

The survey aims to assess the aspirations, expectations, and perceived challenges of prospective fellows and host centers for an ESTES Trauma Traveling Fellowship, guided by CFIR and RE-AIM/PRISM constructs.

Survey Length

5-10 minutes

Data

Data collected by this survey will be anonymously collected and stored on a secure server. The investigator is the Visceral Trauma Section (VTS) of ESTES. The survey responses will inform a peer-reviewed publication.

Do you consent to take part in the survey?

(YES/NO)

Demographics

1. What is your age? (open-ended response)
2. What is your sex? (Male; Female; Prefer not to say; Other (please specify))
3. What is your role in the hospital? (Resident in-training; Attending/Consultant; Other (please specify))
4. Which best describes your career stage? (Surgical resident; Early career (1–5 years post-training); Mid-career (6–15 years post-training); Late career (>15 years post-training))
5. What year did you graduate from medical school? (open-ended response)
6. If you are a in surgical training, what year do you expect to complete it? (open-ended response)
7. If you have completed surgical training, what year did you complete it? (open-ended response)
8. What is/are your surgical subspecialty/ies? (Select all that apply)	(Trauma Surgery; Orthopaedic Surgery; Emergency General Surgery; Other (please specify))
9. In which country do you primarily practice? (open-ended response)
10. What is the name of your Centre / Institution / Hospital? (open-ended response)
11. Do you currently have access to externally funded visiting rotations/fellowships?	(yes; no; please comment)
12. Have you previously undertaken an externally funded rotation/fellowship? (yes; no; if yes, please comment)
13. What would be the ideal duration of a Trauma Traveling Fellowship? (1 - 2 weeks; 3 - 4 weeks; 1 - 2 months; 3 - 6 months; 6 - 12 months)
14. Would you be interested in applying for an ESTES Trauma Traveling Fellowship? (yes; no, but I think it is an interesting idea; no, and I do not think it is necessary) [*If answered yes, then questions 15-19 appear. If answered no, skip to question 20]

Fellow Aspirations

15. Fellowship Aspirations: Which experiences would you hope to gain as a Traveling Fellow? (for each, select either strongly disagree; disagree; neutral; agree; or strongly agree)
    a. Observing trauma resuscitations and/or operations
    b. Participating in clinical activities (resuscitations, operations, ICU)
    c. Conducting or contributing to a research project
    d. Engaging in academic teaching/learning opportunities –
    e. Other (please specify)
16. Key Challenges: What do you foresee as the greatest barriers to attending a Traveling Fellowship? (for each, select either strongly disagree; disagree; neutral; agree; or strongly agree)
    a. Securing study leave approval
    b. Funding travel and accommodation
    c. Visa/insurance requirements
    d. Institutional or licensing permissions for clinical participation
    e. Other (please specify)
17. Funding: Could you personally (or could your institution) contribute funds to support such a Fellowship? (yes; no; if yes, please describe the approximate level or source of support)
18. Preferred sites: Which top three European trauma centres would you most like to visit? (free text; open-ended response)
19. Preferred sites: Optional: Which top three non-European centres would you prioritise? (free text; open-ended response)

Host Expectations

20. Would your centre/hospital be willing to host a Trauma Traveling Fellow? (yes; no, but would be interesting in other centres; no, and I do not think it is necessary) [*If answered yes, then questions 21-23 appear. If answered no, skip to question 24]
21. Hosting Expectations: Which opportunities could your centre realistically offer to a Traveling Fellow? (for each, select either strongly disagree; disagree; neutral; agree; or strongly agree)
    a. Observational access to trauma operations, resuscitations, ICU
    b. Participation in local teaching/academic meetings
    c. Guidance or collaboration on research
    d. Assistance with necessary observer or clinical-participation permissions
    e. To actively participate in clinical/patient activities (operations, resuscitations, ICU
    f. Other (please specify)
22. Hosting Barriers: What potential challenges might prevent your centre from hosting a Traveling Fellow? (for each, select either strongly disagree; disagree; neutral; agree; or strongly agree)
    a. Securing clinical privileges or legal permissions
    b. Ensuring adequate supervision and teaching staff
    c. Competition with local trainees for operative or learning experiences
    d. Limited resources (funding, space)
    e. Limited clinical activity to make a Fellowship worthwhile
    f. Other (please specify)
23. Financial Support: Does your hospital or department have any capacity to sponsor or partially fund a Traveling Fellow? (yes; no; if yes, please describe the approximate level or source of support. (free text)

CFIR

24. Please rate your agreement with the following statements (for each, select either strongly disagree (strong barrier); disagree (weak barrier); neutral; agree (weak facilitator); or strongly agree (strong facilitator))
    a. “The Traveling Fellowship aligns with ESTES leadership goals and priorities.”
    b. “The Traveling Fellowship is a needed initiative for residents and/or early career surgeons.”
    c. “It is advantageous for ESTES to implement the Traveling Fellowship rather than an alternative organisation.”
    d. “ESTES has sufficient resources (time, staff, funding) to implement a Traveling Fellowship.”
    e. “We regularly use performance or feedback data to refine clinical programs."
    f. “I have adequate lines of communication with key stakeholders at ESTES regarding this initiative.”
    g. Other (please specify)

PRISM

25. Please rate your agreement with the following statements (for each, select either not at all; slightly; somewhat; moderately; largely; completely)
    a. Individual Perspective: How well would an ESTES Trauma Traveling Fellowship align with potential fellows’ expectations?
    b. Host/Organisational Perspective: How well would an ESTES Trauma Traveling Fellowship align with the expectations of the Host Centres?
    c. Implementation and Sustainability Infrastructure: How well would an ESTES Trauma Traveling Fellowship align with the available resources, staff, workflow, responsibilities and support functions of Host Centres?
    d. External Environment: How well would an ESTES Trauma Traveling Fellowship align with the characteristics of European trauma clinical practice? (e.g., policies, guidelines, norms)
    e. Optional free text: “Any additional comments on feasibility, implementation, sustainability, or equity?”

RE-AIM

26. Please rate your agreement with the following statements (for each, select either not at all; slightly; somewhat; moderately; largely; completely)
    a. Reach to Host Centres: “How likely is it that a high percentage of invited host centers (including those with limited resources) will host a Fellow?”
    b. Reach to Fellows: “How likely is it that a high percentage of eligible fellows (including those with limited resources) will apply and complete a Fellowship?”
    c. Effectiveness: “How likely is it that this Fellowship will measurably improve trauma training or surgical practice for Fellows?”
    d. Adoption by Host Centres: “How likely is it that a wide variety of centres (including those with limited resources) will adopt this Fellowship as Host Centres?”
    e. Adoption by Fellows: “How likely is it that a wide variety of fellows (including those with limited resources) will apply for this Fellowship?”
    f. Implementation: “How likely is it that each participating site will consistently offer core Trauma Fellowship experiences (e.g., resuscitations, operations, etc.)?”
    g. Maintenance: “How likely is it that the Trauma Fellowship will be sustained over at least 1–2 years?”
    h. Optional free text: “Any additional comments on reach, efficacy, adoption, implementation and maintenance?”

## APPENDIX: Checklist for Reporting Results of Internet E-Surveys (CHERRIES)

| Checklist Item | Explanation | Page Number |
| --- | --- | --- |
| Describe survey design | Describe target population, sample frame. Is the sample a convenience sample? (In “open” surveys this is most likely.) | 4 |
| IRB approval | Mention whether the study has been approved by an IRB. | 4 |
| Informed consent | Describe the informed consent process. Where were the participants told the length of time of the survey, which data were stored and where and for how long, who the investigator was, and the purpose of the study? | 4 |
| Data protection | If any personal information was collected or stored, describe what mechanisms were used to protect unauthorized access. | 4 |
| Development and testing | State how the survey was developed, including whether the usability and technical functionality of the electronic questionnaire had been tested before fielding the questionnaire. | 4 |
| Open survey versus closed survey | An “open survey” is a survey open for each visitor of a site, while a closed survey is only open to a sample which the investigator knows (password-protected survey). | 4 |
| Contact mode | Indicate whether or not the initial contact with the potential participants was made on the Internet. (Investigators may also send out questionnaires by mail and allow for Web-based data entry.) | 4 |
| Advertising the survey | How/where was the survey announced or advertised? Some examples are offline media (newspapers), or online (mailing lists – If yes, which ones?) or banner ads (Where were these banner ads posted and what did they look like?). It is important to know the wording of the announcement as it will heavily influence who chooses to participate. Ideally the survey announcement should be published as an appendix. | 4 |
| Web/E-mail | State the type of e-survey (e.g., one posted on a Web site, or one sent out through e-mail). If it is an e-mail survey, were the responses entered manually into a database, or was there an automatic method for capturing responses? | 4 |
| Context | Describe the Web site (for mailing list/newsgroup) in which the survey was posted. What is the Web site about, who is visiting it, what are visitors normally looking for? Discuss to what degree the content of the Web site could pre-select the sample or influence the results. For example, a survey about vaccination on an anti-immunization Web site will have different results from a Web survey conducted on a government Web site | 4 |
| Mandatory/voluntary | Was it a mandatory survey to be filled in by every visitor who wanted to enter the Web site, or was it a voluntary survey? | 4 |
| Incentives | Were any incentives offered (e.g., monetary, prizes, or non-monetary incentives such as an offer to provide the survey results)? | 4 |
| Time/Date | In what timeframe were the data collected? | 4 |
| Randomization of items or questionnaires | To prevent biases items can be randomized or alternated. | NA |
| Adaptive questioning | Use adaptive questioning (certain items, or only conditionally displayed based on responses to other items) to reduce number and complexity of the questions. | NA |
| Number of Items | What was the number of questionnaire items per page? The number of items is an important factor for the completion rate. | 4 and supplementary material |
| Number of screens (pages) | Over how many pages was the questionnaire distributed? The number of items is an important factor for the completion rate. | 4 |
| Completeness check | It is technically possible to do consistency or completeness checks before the questionnaire is submitted. Was this done, and if “yes”, how (usually JAVAScript)? An alternative is to check for completeness after the questionnaire has been submitted (and highlight mandatory items). If this has been done, it should be reported. All items should provide a non-response option such as “not applicable” or “rather not say”, and selection of one response option should be enforced. | NA |
| Review step | State whether respondents were able to review and change their answers (e.g., through a Back button or a Review step which displays a summary of the responses and asks the respondents if they are correct). | NA |
| Unique site visitor | If you provide view rates or participation rates, you need to define how you determined a unique visitor. There are different techniques available, based on IP addresses or cookies or both. | NA |
| View rate (Ratio of unique survey visitors/unique site visitors) | Requires counting unique visitors to the first page of the survey, divided by the number of unique site visitors (not page views!). It is not unusual to have view rates of less than 0.1 % if the survey is voluntary. | NA |
| Participation rate (Ratio of unique visitors who agreed to participate/unique first survey page visitors) | Count the unique number of people who filled in the first survey page (or agreed to participate, for example by checking a checkbox), divided by visitors who visit the first page of the survey (or the informed consents page, if present). This can also be called “recruitment” rate. | NA |
| Completion rate (Ratio of users who finished the survey/users who agreed to participate) | The number of people submitting the last questionnaire page, divided by the number of people who agreed to participate (or submitted the first survey page). This is only relevant if there is a separate “informed consent” page or if the survey goes over several pages. This is a measure for attrition. Note that “completion” can involve leaving questionnaire items blank. This is not a measure for how completely questionnaires were filled in. (If you need a measure for this, use the word “completeness rate”.) | 4 |
| Cookies used | Indicate whether cookies were used to assign a unique user identifier to each client computer. If so, mention the page on which the cookie was set and read, and how long the cookie was valid. Were duplicate entries avoided by preventing users access to the survey twice; or were duplicate database entries having the same user ID eliminated before analysis? In the latter case, which entries were kept for analysis (e.g., the first entry or the most recent)? | NA |
| IP check | Indicate whether the IP address of the client computer was used to identify potential duplicate entries from the same user. If so, mention the period of time for which no two entries from the same IP address were allowed (e.g., 24 hours). Were duplicate entries avoided by preventing users with the same IP address access to the survey twice; or were duplicate database entries having the same IP address within a given period of time eliminated before analysis? If the latter, which entries were kept for analysis (e.g., the first entry or the most recent)? | NA |
| Log file analysis | Indicate whether other techniques to analyse the log file for identification of multiple entries were used. If so, please describe. | NA |
| Registration | In “closed” (non-open) surveys, users need to login first and it is easier to prevent duplicate entries from the same user. Describe how this was done. For example, was the survey never displayed a second time once the user had filled it in, or was the username stored together with the survey results and later eliminated? If the latter, which entries were kept for analysis (e.g., the first entry or the most recent)? | 4 |
| Handling of incomplete questionnaires | Were only completed questionnaires analysed? Were questionnaires which terminated early (where, for example, users did not go through all questionnaire pages) also analysed? | 4 |
| Questionnaires submitted with an atypical timestamp | Some investigators may measure the time people needed to fill in a questionnaire and exclude questionnaires that were submitted too soon. Specify the timeframe that was used as a cut-off point, and describe how this point was determined. | NA |
| Statistical correction | Indicate whether any methods such as weighting of items or propensity scores have been used to adjust for the non-representative sample; if so, please describe the methods. | NA |

This checklist has been modified from Eysenbach G. Improving the quality of Web surveys: the Checklist for Reporting Results of Internet E-Surveys (CHERRIES). J Med Internet Res. 2004 Sep 29;6(3):e34 [erratum in J Med Internet Res. 2012; 14(1): e8.]. Article available at [https://www.jmir.org/2004/3/e34](https://www.jmir.org/2004/3/e34/)/; erratum available <https://www.jmir.org/2012/1/e8/>. Copyright ©Gunther Eysenbach. Originally published in the [Journal of Medical Internet](http://www.jmir.org) Research, 29.9.2004 and 04.01.2012.

This is an open-access article distributed under the terms of the Creative Commons Attribution License (<https://creativecommons.org/licenses/by/2.0/>), which permits unrestricted use, distribution, and reproduction in any medium, provided the original work, first published in the Journal of Medical Internet Research, is properly cited.
